# Supplementary material for: Can Nutritional Intervention for Obesity and Comorbidities Slow Down Age-Related Hearing Impairment?
Source: Nutrients. 2019 Jul 21;11(7):1668. doi: 10.3390/nu11071668 (PMC6682960; doi:10.3390/nu11071668)
Supplement: Supplementary file 1 [file nutrients-11-01668-s001.pdf]

Table S1	Studies included in this review				
**Nutrient**	**Study subjects**	**Hearing test method**	**Study design**	**Main findings**	**Author and year**
Low fat diet	C57BL/6J mice CBA/N-slc mice	ABR	Experimental study design	High-fat food delays ARHI. The antioxidant of vitamin E is rich in the high fat diet.	Fujita et al., 2015 [58]
	Sprague-Dawley rats	ABR	Experimental study design	High fat diet may induce oxidative stress, mitochondrial damage, and apoptosis in the inner ear which increases risk of ARHI.	Du et al., 2012 [55]
	CBA/CaJ mouse	ABR, DPOAEs	Experimental study design	Induction of diabetes significantly accelerates hearing impairment.	Vasilyeva et al., 2009 [56]
	CD/1 mice	ABR	Experimental study design	Diet-induced obesity exacerbates hearing degeneration via increased hypoxia, inflammatory responses, and apoptosis signaling pathways.	Hwang et al., 2013 [57]
	Humans (aged ≥50 y)	PTA	Retrospective cross-sectional analysis	Dietary intake of cholesterol is associated with progression of ARHI.	Gopinath et al., 2011 [59]
Statins	Humans (aged ≥50 y)	PTA	Retrospective cross-sectional analysis	Statins may have beneficial influence on preventing ARHI.	Gopinath et al., 2010 [74]

	C57BL/6J mice ApoE-deficient mice	ABR DPOAEs	Experimental study design	Statins could slow down ARHI by improving blood flow of inner ear	Syka et al., 2007 [61]
Aldosterone	Humans (aged ≥58y)	PTA, TEOAEs HINT	Case control study	Serum aldosterone level is lower in ARHI. Aldosterone may have protective effect on ARHI.	Tadros et al., 2005 [67]
	CBA/CaJ mice	ABR	Experimental study design	Protective effect of aldosterone on ARHI is via cellular stabilization in cochlear lateral wall cells.	Halonen et al., 2016 [66]
n-3 PUFAs	C57BL/6J mice	ABR, DPOAEs	Experimental study design	Long-term protective role of n-3 PUFAs on cochlear homocysteine metabolism and progression of ARHI.	Martínez et al., 2015 [73]
	Humans (aged ≥50 y)	PTA	Cross-section and longitudinal prospective design	n-3 PUFAs in the diet could be beneficial to preserve cochlear function and reduce ARHI.	Gopinath et al., 2010 [74]
	Humans (aged 50-70 y)	PTA	Cross-section and longitudinal prospective design	Inverse association between plasma very long-chain n-3 PUFAs and hearing loss.	Dullemeijer C et al., 2010 [75]
Alpha-lipoic acid	Fischer rats	ABR	Experimental study design	Alpha-lipoic acid repairs age-induced cochlea mitochondrial damage and reduces ARHI	Seidman et al., 2005 [76]
Lecithin	Fischer rats	ABR	Experimental study design	Lecithin may preserve cochlear mitochondrial function and reduced ARHI.	Seidman et al., 2002 [78]

Tea	Human (aged ≥55 y)	PTA	Retrospective cohort study	Oolong tea drinking is associated with better central auditory function in the aged.	Hwang et al., 2012 [80]
Ginseng	C57BL/6 mice	ABR	Experimental study design	Ginseng has protective effect on ARHI.	Tian et al., 2014 [83]
	C57BL/KsJ mice	ABR<br>AMLR<br>TEOAEs	Experimental study design	Ginseng could reduce hearing loss by improving insulin sensitivity.	Hong et al., 2013 [84]

ABR: auditory brainstem response; ARHI: age-related hearing impairment; DPOAEs: distortion product otoacoustic emissions; n-3 PUFA: omega-3 polyunsaturated fatty acids; HINT: Hearing in noise test; PTA: pure-tone audiometry; AMLR: auditory middle latency response; TEOAEs: transient evoked otoacoustic emissions
